# Exploring Client Preferences for Psychological Counselors in a Chinese Online Health Community: Longitudinal Study

**DOI:** 10.2196/58428

**Published:** 2024-10-10

**Authors:** Xiandong Feng, Yinhuan Hu, Holger Pfaff, Sha Liu, Jinzhu Xie, Zemiao Zhang

**Affiliations:** 1 School of Medicine and Health Management, Tongji Medical College Huazhong University of Science and Technology Wuhan China; 2 Major Disciplinary Platform under Double First-Class Initiative for Liberal Arts at Huazhong University of Science and Technology (Research Center for High-Quality Development of Hospitals) Wuhan China; 3 Institute of Medical Sociology, Health Services Research, and Rehabilitation Science, Faculty of Human Sciences & Faculty of Medicine, University of Cologne Cologne Germany; 4 Hubei Third People's Hospital Wuhan China

**Keywords:** signaling theory, psychological counselor, online health communities, clients’ choice

## Abstract

**Background:**

Although online health communities are acknowledged for their role in bridging the supply-demand gap in mental health services, the client decision-making process in these environments remains underexplored.

**Objective:**

This study aimed to explore the impact of different signals presented on psychological counselors’ home pages on clients’ choices.

**Methods:**

Adopting signaling theory as the framework, this study classified information into online and offline signals and developed a theoretical model to examine client choice behaviors. We collected data from 487 psychological counselors in a leading Chinese online mental health community during March, June, September, and December 2023. Based on these data, we constructed a 4-period balanced panel dataset. A fixed effects model was used to analyze which signals influence clients’ choices of psychological counselors.

**Results:**

Regarding online signals, the service price (β=0.186, *P*<.001) and online reputation (β=0.489, *P*=.002) of psychological counselors positively influence clients’ choices. Concerning offline signals, psychological counselors’ practical experience (β=0.007, *P*<.001) is positively related to clients’ choices. Moreover, the results indicate that the relationship between a counselor’s prosocial behavior and clients’ choices is not linear but rather exhibits an inverted U-shape.

**Conclusions:**

This study reveals that the varied information provided by psychological counselors has distinct impacts on clients’ choices in online health communities. It broadens the application of signaling theory to online behaviors and emphasizes the importance of both online and offline signals. These insights offer strategic guidance for counselors and online platforms to better meet potential clients’ needs by optimizing the information presented on psychological counselors’ home pages.

## Introduction

### Background

Mental health constitutes a fundamental aspect of overall health and is recognized as a basic human right [1]. According to a report from the World Health Organization (WHO), an estimated 970 million people worldwide have mental disorders, such as depression, anxiety, and eating disorders, creating a substantial demand for mental health services [2]. However, nearly half of the global population resided in countries where there was only 1 psychiatrist available for every 200,000 individuals or more [2]. The severe imbalance between the supply and demand for mental health professionals poses a significant challenge to global public health.

The development of Web 2.0 has facilitated the emergence of online health communities (OHCs), greatly bridging the accessibility gap in mental health services [3]. These platforms serve as a novel channel for individuals to access information and emotional support [4], aligning with the delivery of mental health services. First, compared to physical diseases, mental health issues are less tangible and can often be mitigated through psychological counseling [5,6]. Online counseling, a central component of OHCs, uses video, voice, and text to overcome geographical and temporal barriers [7], thereby enhancing the accessibility of mental health services. Second, the societal stigma associated with mental health issues may cause individuals to avoid professional health services or hesitate to seek help [8]. Online counseling presents an advantage in this context by ensuring anonymity, enabling individuals to undergo counseling privately without the fear of recognition by acquaintances.

OHCs for mental health have been increasingly acknowledged and used, especially in emerging economies like China, where medical resources are limited but internet access is prevalent [9]. In recent years, driven by a significant increase in demand, OHCs for psychological counseling in China have experienced rapid development. According to the China Internet Network Information Center, by the end of 2023, the number of internet users in China had exceeded 1 billion, providing a solid foundation for the promotion of OHC services [10]. Moreover, the cultural background and social structure in China differ from those in Western countries, which may influence the acceptance and implementation of online psychological counseling [11]. For example, mental health issues carry a significant stigma in Chinese society, making the anonymity of online counseling more appealing [12]. In addition, mental health resources in China are unevenly distributed, particularly in rural and remote areas where these resources are scarce [13]. The advancement of internet technology has enabled OHCs to provide convenient mental health services to residents in these areas, making them particularly relevant in the Chinese context [14]. Thus, choosing China as the focus of this study not only helps to reveal unique phenomena in the field of OHCs in China but also provides experience and references for the global development of online psychological counseling.

Unlike offline services, online services frequently occur between parties lacking any prior transaction history [15]. This absence of prior interaction usually leaves consumers without the opportunity to evaluate or trial the service, thereby compelling them to assume the “risk of prior performance” [16]. In the health care sector, information asymmetry is more marked: whereas health professionals understand the characteristics of their services, consumers possess limited insight [17]. Therefore, the decision-making processes of individuals in selecting among numerous online psychological counselors have become a critical issue. Signaling theory provides a theoretical framework for understanding this information asymmetry and suggests potential solutions [18]. According to signaling theory, information asymmetry can be mitigated when the parties with more information share some signals with the other parties, and the recipient of these signals can interpret them and adjust their behavior accordingly [19]. Within the environment of OHCs, health care providers disclose information to potential users, such as price and reputation, and people can use this information to make what they perceive to be the appropriate choice for their situations [20].

### Objective

Existing research has demonstrated the positive impact of online psychological counseling. For example, studies have shown that online counseling can significantly improve clients’ emotional and behavioral issues [7,21-23]. However, little research addresses the decision-making process clients undergo when selecting online counselors. At the same time, although previous studies have demonstrated that online information in OHCs can influence consumer choices, few have applied signaling theory to online psychological counseling. In addition, given that OHCs cover a wide range of diseases, the needs and preferences of signal receivers may vary [20]. Therefore, the research question for this study is as follows: What factors influence clients’ choice of counselors in OHCs? To answer this question, this paper constructs a theoretical model based on signaling theory. Furthermore, we chose to study both online and offline signals to systematically identify the factors that influence clients’ choices of online psychological counselors.

### Research Framework and Hypothesis Development

#### Signaling Theory

Signaling theory aims to reduce information asymmetries among different parties across varied socioeconomic contexts [18]. This theoretical framework is composed of 3 core elements: the signaler (service provider), the receiver (consumer), and the signal itself [24]. In the context of the online service market, signals are divided into online and offline categories, thereby providing a mechanism for users to evaluate service quality [25]. Online signals originate from virtual consultation–based platforms, including factors like price [26], prosocial behaviors [27,28], and online reputation [29,30]. Offline signals derive from sellers’ attributes [31], such as professional titles [32,33], practical experience [34], and education level [35].

Within OHCs, the disparity in knowledge between professionals and clients is profound, making signaling particularly vital [36]. Research has demonstrated the impact of different signals on clients in OHCs. For instance, Deng et al [3] found that online effort and reputation of physicians greatly influence patients’ decisions on the Good Doctor website. Hsu et al [37] observed that the offline promotion of physicians positively affects patient choices in OHCs and leads them to give higher online ratings. Moreover, Zhang et al [4] found that physicians’ offline expertise negatively affects user adoption decisions, while online experience has a positive impact. This study builds on existing research by applying signaling theory to the context of psychological counseling within OHCs, examining how different signals drive clients’ choices in online mental health services.

#### Price

In the framework of signaling theory, price is regarded as a crucial signal of product quality, particularly for intangible services such as health care [38]. Unlike traditional health care services with pricing set by institutions or governments, OHCs allow psychological counselors to set their service prices [39]. This market-driven pricing strategy helps eliminate less competent providers, encouraging the rest to enhance their service quality [40].

Consumers often struggle to assess service quality and rely on price as a quality indicator [41] due to a prevalent belief in market economies that associates high prices with superior quality and greater consumption value. Numerous studies show that despite viewing price as a sacrifice, consumers tend to pay more for what they deem reasonable pricing, especially when purchasing intangible services that are costly, infrequently bought, and require complex decision-making [39,42,43]. Therefore, we propose the following hypothesis:

- H1: The psychological counselors’ service price is positively related to clients’ choices.

#### Prosocial Behavior

Prosocial behavior, also known as altruism, refers to actions that align with societal expectations, where the performer willingly engages in these behaviors despite lacking apparent personal benefits [44-46]. Health professionals’ prosocial behavior can manifest through diverse actions, such as providing free answers to patients’ questions, sharing knowledge articles, and transparently sharing diagnostic and treatment details [47]. These behaviors influence doctor-patient trust [48,49], financial rewards [25,50], and patient satisfaction, among other outcomes [37,51].

Previous research suggests that it is unlikely for a psychological counselor’s prosocial behavior to have a consistent linear relationship with clients’ selection criteria [52]. In this study, excessive prosocial behavior refers to situations where the counselor’s actions exceed typical expectations of helpfulness and altruism, potentially leading clients to question the genuineness and motives behind these behaviors. In other words, when a counselor exhibits such excessive prosocial behavior, clients may doubt the intent and authenticity of these actions. This is consistent with the signaling theory, which posits that signals intended to convey quality or trustworthiness can lose their effectiveness if perceived as exaggerated or insincere. For example, clients might doubt whether the prosocial behavior displayed on the website is genuine or if it serves as a marketing strategy [53]. Consequently, we propose the following hypothesis:

- H2: There exists a reverted U-shaped relationship between psychological counselors’ prosocial behavior and clients’ choices.

#### Online Reputation

Online reputation is a complex and dynamic concept that reflects the past behavior of individuals or organizations [54,55]. It empowers consumers to peruse and assess the experiences and reviews of others, thereby enhancing their ability to accurately ascertain the quality of a product or service [56,57]. Online reputation appears in many forms, including product or service ratings, reviews, testimonials, and records of the seller’s transaction history and performance [58].

Research has confirmed that the online reputation of individuals and organizations significantly influences consumer purchasing behavior [36,59]. Given the complexity and professionalism of health information, consumers often rely on others’ experiences and evaluations when making decisions [60]. Therefore, we propose the following hypothesis:

- H3: The psychological counselors’ online reputation is positively related to clients’ choices.

**Psychological Counselor’s Characteristics**

In traditional health care environments, clients typically evaluate a service provider’s professional competence and technical skills based on personal characteristics such as professional titles, education level, and practical experience [61-64].

First, from the consumer’s perspective, a service provider’s professional titles often endorse their professionalism [34]. In situations with information asymmetry, the professional titles granted by authoritative organizations are considered a reliable competence indicator. Second, management studies have shown that experience is crucial for improving work effectiveness and customer satisfaction [48]. Therefore, practical experience reflects a psychological counselor’s competence, as longer tenure often indicates better problem-solving abilities. Finally, education significantly influences clients’ choices of psychological counselors. As mental health is interdisciplinary, higher-educated counselors are perceived as more adept at handling complex psychological issues. Therefore, we propose the following hypothesis:

- H4a: The psychological counselors’ professional titles are positively related to clients’ choices.
- H4b: The psychological counselors’ practical experience is positively related to clients’ choices.
- H4c: The psychological counselors’ education level is positively related to clients’ choices.

**Figure 1:**
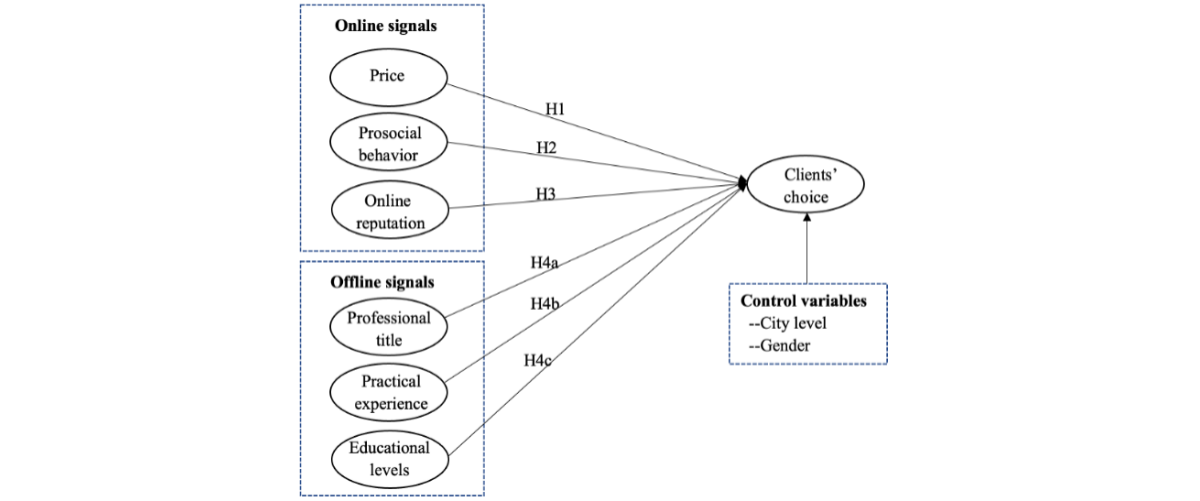
Hypotheses and research model.

Based on the above assumptions, we construct a research model, as shown in Figure 1.

## Methods

### Research Context

**Figure 2:**
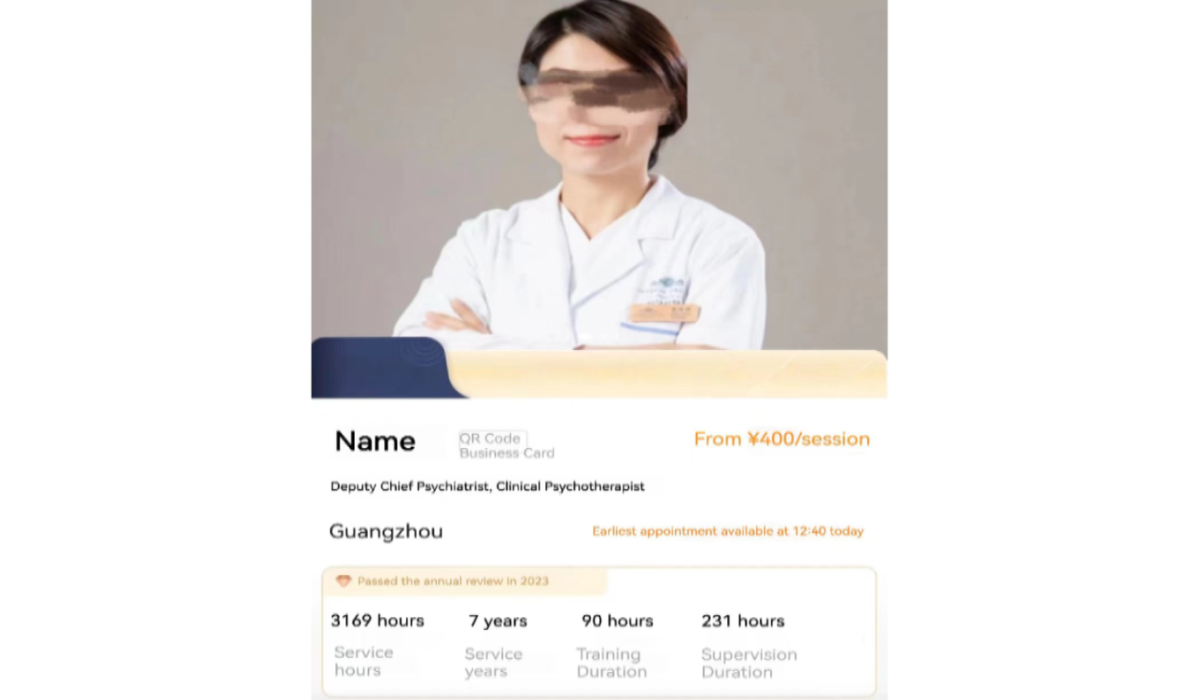
Sample of a psychological counselor’s home page.

**Figure 3:**
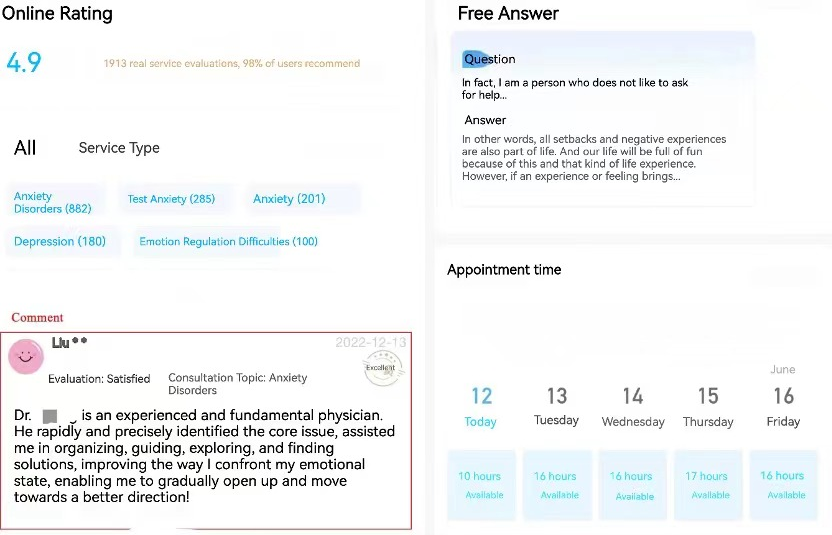
Details of a psychological counselor.

We selected YiDianLing as our data sampling community for several reasons. First, YiDianLing is one of the largest OHCs in China dedicated to mental health, with approximately 39 million registered users and 42,000 resident professional psychological counselors. This extensive user base ensures a diverse and comprehensive dataset, which increases the generalizability of the results. Second, the platform’s services are available nationwide, providing a broad geographic representation of clients and counselors from different regions across China. This geographic diversity further enhances the generalizability of our study’s findings within the context of the Chinese mental health landscape. Third, YiDianLing offers both chargeable services and free consultations, providing data to support our research on the prosocial behavior of psychological counselors. Fourth, YiDianLing maintains high certification standards for mental health service providers. All applicants must submit relevant offline information, and only those who meet the platform’s certification criteria can establish their home pages. This ensures the reliability and professionalism of the counselors included in the study. Fifth, psychological counselors registered on YiDianLing have personal home pages that contain quantitative data relevant to this study, such as the total number of consultations, service price, and free consultations. Figures 2 and 3 provide detailed views of a counselor’s home page. Therefore, YiDianLing provides a robust and reliable data source that aligns well with the objectives of our research.

### Data Collection

We developed a web crawler program to collect information from the personal home pages of psychological counselors on YiDianLing. Although there are many registered counselors on YiDianLing, the platform only showcases approximately the top 1000 active counselors. To ensure the representativeness of our study subjects, we used a 3-step selection process. First, the counselors must have provided services to at least 1 client on the platform. Second, these counselors must have available services for booking within the next 5 days to confirm their active status on the platform. Finally, their information on the home pages had to be complete and include all the target data for our research. Based on these criteria, we initially identified 667 counselors on the YiDianLing platform as our research sample.

To further ensure the validity of the sample data, we collected profile information of the same counselors at 4 different time points (March, June, September, and December 2023). This phased data collection method allowed us to account for potential time-related effects. It enabled us to capture a more comprehensive picture of user behavior and service demand dynamics, avoiding the randomness and partiality associated with data from a single time point.

Following data collection, we conducted comprehensive data cleaning and preprocessing to eliminate missing and duplicate data. Ultimately, we reduced the initial sample of 667 counselors to 487 psychological counselors as the final sample. We used this final sample to create a balanced 4-period panel dataset.

### Ethical Considerations

The data for this study were sourced from publicly accessible information available to all users of the online platform. We did not collect any personal information about the clients and anonymized data about counselors. The study was approved by the Ethics Committee of Tongji Medical College of Huazhong University of Science & Technology (IORG0003571).

### Variables

#### Dependent Variable: Clients’ Choices

We measured clients’ choices by the total number of consultations per counselor. This variable was selected as it serves as a robust indicator of clients’ choices of counselors. Specifically, if a counselor has a high total number of consultations, this may indicate that they have a high popularity among clients.

#### Independent Variables

##### Price

We chose the price per service as the measure for the independent variable of price. The price per service is typically prominently displayed on the counselor’s home page, potentially forming the client’s first impression of the counselor’s service cost.

##### Prosocial Behavior

According to the definition of prosocial behavior, we used the number of questions answered for free by counselors as its indicator. This action reflects the counselor’s willingness to share their professional knowledge and offer help without any direct financial return.

##### Online Reputation

The counselor’s reputation was represented by a system-generated rating, computed from feedback provided by all previous clients who have used the counselor’s services. The online rating system operates on a scale from 0 to 5, accurate to 1 decimal place.

##### Professional Titles

In China, there are 3 primary professional titles associated with psychological counselors: counselor, psychotherapist, and psychiatrist. Each of these titles is further categorized into 3 levels: junior, intermediate, and senior. For the purpose of our study, we assigned numerical codes to these levels: 1 for junior, 2 for intermediate, and 3 for senior. As a counselor may hold multiple titles, the variable *professional titles* or *PT* we used in our analysis represented the sum of the codes for all the titles held by the counselor:

PT = PTcounselor + PTpsychotherapist + PTpsychiatrist

##### Practical Experience

Practical experience can be reflected by the number of hours of service, which is directly extracted from the counselor’s home page and measured in hours. Although the number of hours of service is displayed online, it primarily reflects the service duration proof provided by the counselor when registering on the platform. Therefore, we classified practical experience as an offline signal, as it mainly showcases the counselor’s professional experience outside the online platform.

##### Educational Level

The educational levels of counselors were classified into 4 categories and assigned corresponding numerical codes. The categories and codes used were as follows: “1 for college and below,” “2 for a bachelor’s degree,” “3 for a master’s degree,” and “4 for a doctorate and above.”

#### Control Variables

##### City Level

The variability in internet coverage across China’s vast regions could affect clients’ behavior in choosing online psychological counselors. Due to the stigma associated with mental health issues, the platform does not display clients’ personal information. To control for regional differences, we included the city tier of the psychological counselors as a control variable. According to the latest Chinese city classification standards [65], we coded city level classes into 3 categories: “3 for first-tier cities,” “2 for second-tier cities,” and “1 for others.”

##### Gender

Gender was coded as “1 for male” and “2 for female.”

#### Estimation Model

The descriptive statistics for all variables (see Table 1) reveal that clients’ choices, price, prosocial behavior, and practical experience exhibit significantly larger extremum and SD. Such variability might result in biased and inefficient model estimations. Thus, we choose to log-transform these variables to mitigate the impact of extreme values in our model construction.

**Table 1:** Summary statistics.

Variable	Value, mean (SD)	Value, minimum	Value, maximum
CC^a^	2075.21 (2569.61)	1	14,408
Pr^b^	317.38 (248.57)	10	1600
PB^c^	276.44 (223.95)	10	990
OR^d^	4.90 (0.16)	3	5
PT^e^	2.20 (0.98)	0	7
PE^f^	2084.87 (2565.08)	0	14,552
EL^g^	1.59 (0.91)	1	4
CL^h^	2.27 (0.83)	1	3
Gen^i^	1.75 (0.43)	1	2

^a^CC: clients’ choices.

^b^Pr: price.

^c^PB: prosocial behavior.

^d^OR: online reputation.

^e^PT: professional titles.

^f^PE: practical experience.

^g^EL: educational level.

^h^CL: city level.

^i^Gen: gender.

In order to control for unobservable heterogeneity that might influence clients’ choices, we used a dynamic panel data model for our analysis. We used the Hausman test to contrast the fixed and random effects models. Given that the resulting *P* value was below the conventional level of significance (.05), we opted for a fixed effects model for our subsequent analysis. The advantage of the fixed effects model is that it controls for time-invariant characteristics of individuals, allowing us to isolate the effects of variables that change over time. The empirical model is presented as follows:

log(CCit) = β0 + β1log(Prit) + β2log(PBit) + β3[(log(PBit))2] + β4(ORit) + β5(PTit) + β6log(PEit) + β7(ELit) + β8(CLit) + β9(Genit) + λt + νi + εit

where *i* is the index of the counselor and *t* represents the time point. β_1_ to β_9_ are the parameters to be estimated. λ*_t_* represents the time fixed effects, which controls for common changes in mental health needs over time (eg, seasonal variations, holidays). ν*_i_* represents the individual fixed effect, which controls for the stable characteristics of each counselor that do not change over time (eg, registration duration on the platform, counseling style). ε is the error term. *CC* is clients’ choices, *Pr* is price, *PB* is prosocial behavior, *OR* is online reputation, *PT* is professional titles, *PE* is practical experience, *EL* is educational level, *CL* is city level, and *Gen* is gender.

## Results

### Research Model Results

Table 2 presents the correlation coefficients of all variables for the analysis. The results show that almost all of the independent variables exhibit a correlation with the dependent variable. The correlations among the independent variables are significantly low. Moreover, the variance inflation factor values of the independent and control variables are less than 2, indicating that multicollinearity is not a concern in our model.

Table 3 presents the results of our empirical models. Throughout our analysis, we systematically incorporated variables, culminating in the construction of 4 distinct models. We observed a consistent increase in *R*² from 0.337 in Model 1 to 0.962 in Model 4 with the inclusion of variables. The significance levels of the key variables remained consistent across all models. Therefore, we chose to interpret our research results using the final model.

As shown in Model 4, in the online signals category, both price (β_1_=.186, *P*＜.001) and online reputation (β_4_=.489, *P*=.002) positively affect the clients’ choices, thereby supporting H1 and H3. Based on our previous assumptions, we incorporated prosocial behavior and its quadratic term into the empirical model. We find a significant inverted U-shaped relationship between prosocial behavior and the clients’ choices (β_2_=.190, *P*=.002; β_3_=–.018, *P*=.001). After conducting the fixed effects model analysis, we used the U-shape test estimation algorithm by Lind and Mehlum [66] to verify the stability of the inverted U-shaped relationship. The procedure involved 4 main steps. First, we used the Wald test to assess the significance of the linear and nonlinear combinations of prosocial behaviors. The *P* value of the Wald test was .004, indicating that these variables significantly contributed to the model. Second, we calculated the slope of the curve at the low and high points of prosocial behavior. According to our research data, the low point was 2.40 and the high point was 6.90. The results showed that the slope of prosocial behavior at the low point was 0.102, and at the high point, it was –0.060. Third, we used the likelihood ratio test to verify whether the influence of prosocial behavior on clients’ choices increased monotonically at low levels of prosocial behavior and decreased monotonically at high levels. The *P* value of the likelihood ratio test was .001, indicating that this nonlinear relationship was significant. Fourth, we used the Delta confidence interval test to verify whether the poles of the curve fell within the range of high and low values of prosocial behaviors. The results showed that the poles fell within the interval between the 2 values, indicating that the inverted U-shape was considered stable. Therefore, H2 is supported. In the offline signals category, we discovered that practical experience (β_6_=.911, *P*＜.001) has a positive impact on clients’ choices, supporting H4b. However, the effect of professional titles and educational levels on the clients’ choices was found to be insignificant; thus, H4a and H4c are not supported.

**Table 2:** The correlation coefficient table.

Variable			log(CC^a^)		log(Pr^b^)		log(PB^c^)		OR^d^		PT^e^		log(PE^f^)		EL^g^		CL^h^		Gen^i^
**log(CC)**																			
	*r*	1		0.369		0.276		0.370		0.197		0.957		–0.040		–0.011		0.170	
	*P* value	—^j^		<.001		<.001		<.001		<.001		<.001		.08		.64		<.001	
**log(Pr)**																			
	*r*	0.369		1		0.094		0.103		0.083		0.397		–0.004		0.131		0.098	
	*P* value	<.001		—		<.001		<.001		<.001		<.001		.85		<.001		<.001	
**log(PB)**																			
	*r*	0.276		0.094		1		0.061		0.055		0.247		–0.052		–0.058		0.021	
	*P* value	<.001		<.001		—		.01		.02		<.001		.02		.01		.36	
**OR**																			
	*r*	0.37		0.103		0.061		1		0.095		0.403		–0.079		0.007		0.202	
	*P* value	<.001		<.001		0.01		—		<.001		<.001		<.001		.75		<.001	
**PT**																			
	*r*	0.197		0.083		0.055		0.095		1		0.195		0.054		0.001		0.045	
	*P* value	<.001		<.001		.02		<.001		—		<.001		.02		.99		.049	
**log(PE)**																			
	*r*	0.957		0.397		0.247		0.403		0.195		1		–0.047		0.001		0.200	
	*P* value	<.001		<.001		<.001		<.001		<.001		—		.04		.99		<.001	
**EL**																			
	*r*	–0.040		–0.004		–0.052		–0.079		0.054		–0.047		1		0.042		0.076	
	*P* value	.08		.85		.02		<.001		.02		.04		—		.06		<.001	
**CL**																			
	*r*	–0.011		0.131		–0.058		0.007		0.001		0.001		0.042		1		0.095	
	*P* value	.64		<.001		.01		.75		.99		.99		.06		—		<.001	
**Gen**																			
	*r*	0.170		0.098		0.021		0.202		0.045		0.200		0.076		0.095		1	
	*P* value	<.001		<.001		.36		<.001		.049		<.001		<.001		<.001		—	

^a^CC: clients’ choices.

^b^Pr: price.

^c^PB: prosocial behavior.

^d^OR: online reputation.

^e^PT: professional titles.

^f^PE: practical experience.

^g^EL: educational level.

^h^CL: city level.

^i^Gen: gender.

^j^Not applicable.

**Table 3:** Empirical model results.

Variable	Model 1	*P* value	Model 2	*P* value	Model 3	*P* value	Model 4	*P* value
log(Pr^a^), mean (SE)	—^b^		0.421 (0.154)	.01	—		0.186 (0.042)	<.001
log(PB^c^), mean (SE)	—		0.148 (0.028)	<.001	—		0.190 (0.063)	.002
(log(PB))^2^, mean (SE)	—		—		—		–0.018 (0.006)	.01
OR^d^, mean (SE)	—		9.820 (0.517)	<.001	—		0.489 (0.157)	.002
PT^e^, mean (SE)	—		—		0.027 (0.023)	.24	0.024 (0.023)	.29
log(PE^f^), mean (SE)	—		—		0.920 (0.006)	<.001	0.911 (0.007)	<.001
EL^g^, mean (SE)	—		—		0.005 (0.010)	.63	0.007 (0.010)	.50
CL^h^, mean (SE)	–0.937 (0.488)	<.001	–0.675 (0.216)	.002	–0.291 (0.059)	<.001	–0.267 (0.059)	<.001
Gen^i^, mean (SE)	–2.125 (0.488)	<.001	–2.314 (0.436)	<.001	–0.121 (0.119)	.31	–0.200 (0.119)	.09
Time fixed effect	Yes		Yes		Yes		Yes	
Individual fixed effect	Yes		Yes		Yes		Yes	
*R* ^2^	0.337		0.485		0.961		0.962	
Number of time periods, T	4		4		4		4	
Observations, N	1948		1948		1948		1948	

^a^Pr: price.

^b^Not applicable.

^c^PB: prosocial behavior.

^d^OR: online reputation.

^e^PT: professional titles.

^f^PE: practical experience.

^g^EL: educational level.

^h^CL: city level.

^i^Gen: gender.

### Robustness Check

In the robustness check section, we divided the dataset into 2 groups based on service duration. These two subsamples included (1) psychological counselors with fewer than 1000 hours of service and (2) those with 1000 hours or more. As service duration is consistent within each group, we did not include this factor in the model. Table 4 shows that the findings from these subsamples correspond with the primary results from the full dataset. Therefore, research findings exhibit high robustness and reliably support our preliminary results.

**Table 4:** Robustness check results.

Variable	Model 5 (group 1)	*P* value	Model 6 (group 2)	*P* value
log(Pr^a^), mean (SE)	0.207 (0.333)	<.001	0.497 (0.070)	<.001
log(PB^b^), mean (SE)	1.261(0.342)	<.001	0.331 (0.146)	.02
(log(PB))^2^, mean (SE)	–0.140 (0.036)	<.001	–0.029 (0.014)	.04
OR^c^, mean (SE)	7.866 (0.636)	<.001	4.105 (1.774)	.02
PT^d^, mean (SE)	–0.139 (0.042)	.42	0.028 (0.036)	.44
EL^e^, mean (SE)	0.006 (0.086)	.94	0.004 (0.015)	.04
CL^f^, mean (SE)	–0.672 (0.443)	.13	–0.711 (0.101)	<.001
Gen^g^, mean (SE)	–1.475 (0.539)	.01	–0.377 (0.667)	.27
Time fixed effect	Yes		Yes	
Individual fixed effect	Yes		Yes	
*R* ^2^	0.694		0.177	
Number of time periods, T	4		4	
Observations, N	800		1148	

^a^Pr: price.

^b^PB: prosocial behavior.

^c^OR: online reputation.

^d^PT: professional titles.

^e^PE: practical experience.

^f^EL: educational level.

^g^CL: city level.

^h^Gen: gender.

## Discussion

### Analysis of Results

In this paper, we draw on signaling theory to investigate how information on psychological counselors’ home pages influences clients’ choices. The findings highlight the crucial role of online and offline signals in shaping decision-making processes and demonstrate that effective information disclosure can encourage consumers to select a psychological counselor.

In the context of online signals, it has been found that both price and online reputation affect a client’s choice of psychological counselor, aligning with the findings of previous relevant studies [26,42,43,67-69]. On the one hand, given that consumers might not possess the requisite expertise to evaluate professional services, clients are often willing to pay a premium in exchange for the assurance of higher service quality [39]. As prices increase, consumers expect better services from a counselor, and thus are more inclined to select that counselor. On the other hand, a strong online reputation can boost consumers’ trust in a counselor’s professional skills. Demonstrating an online reputation highlights the consistency and professionalism of a counselor’s service quality and reveals the professional attitude and commitment they maintain during service delivery.

In the context of offline signals, it was determined that practical experience has a positive impact on a client’s choice of a psychological counselor. Offline signals mainly offer insights into a counselor’s personal attributes, thus often considered reflective of their skills and professional standards. Although offline signals originate from a physical environment, this study demonstrates that consumers attribute significant value to them and recognize their essential role in decision making. In other words, even in the digital sphere, the importance and impact of offline signals are preserved.

Interestingly, we discovered an inverted U-shaped relationship between a psychological counselor’s prosocial behavior and clients’ choices. This suggests that while clients appreciate a certain level of prosocial behavior, an overly excessive display may arouse suspicion and negatively affect their choices. Specifically, moderate prosocial behavior can improve a counselor’s image and online reputation, thus enhancing client trust. However, exceeding a certain threshold of prosocial behavior may be perceived as insincere, resulting in increased client skepticism toward the counselor.

### Theoretical Implications

Our study broadens the scope of theoretical research on signaling theory. First, we introduce signaling theory into the specific service domain of online psychological counseling and establish a theoretical framework to clarify the role of online and offline signals in the process of clients choosing a psychological counselor. The results of this study align with Zhou et al [70], which highlights the significance of both demonstration and description signals in online mental health markets and demonstrates how various types of signals can influence clients’ choices.

Second, this study considers the service price, online reputation, and prosocial behavior as signals and incorporates them into the research framework, which is an approach previously unexplored in previous studies. Previous studies have often focused on isolated online signals, such as online reviews and ratings [25,71,72]. Our research integrates both online signals and offline signals, offering a more comprehensive framework that enhances the understanding of how different signals interact to influence clients’ choices.

Finally, our research results reveal an inverted U-shaped relationship between a psychological counselor’s prosocial behavior and client’s choices. This finding addresses a gap in the literature, where prosocial behavior was generally considered to have a linear positive effect [73-76]. Our study shows that there is an optimal level of prosocial behavior, beyond which client skepticism may arise, thus contributing to a more nuanced understanding of the dynamics at play.

### Managerial Implications

For psychological counselors in OHCs, our study reveals the positive influence of service price on clients’ choices. Consequently, psychological counselors should leverage their pricing autonomy in OHCs, using their service price as a signal of their capability to convey accurate quality signals to potential clients. Moreover, considering the significant impact of an online reputation on client’s choice, it is crucial for psychological counselors to carefully manage client relationships and maintain their online reputation. This suggests that, besides providing high-quality services, psychological counselors should proactively respond to client feedback, engage in OHC activities, and enhance their online visibility to gain a competitive edge in the domain of online psychological counseling [77]. Finally, our research exposes an inverted U-shaped relationship between a psychological counselor’s prosocial behavior and clients’ choices. This finding suggests that psychological counselors must adeptly balance and temper their prosocial behavior in practice, aiming for an ideal equilibrium that attracts clients while maintaining a professional image.

For OHC platforms, despite operating in a digital environment, offline signals from psychological counselors still play a significant role in client selection. Therefore, platforms may consider displaying more offline personal information on the counselors’ profile pages, such as their affiliated institutions and accolades, which can enhance information transparency and boost client trust. At the same time, platforms need to recognize the high level of user attention given to key signals such as price, online reputation, and prosocial behavior. As a result, when designing web and app interfaces, platforms should strategically position this information prominently, allowing users to quickly and efficiently access these details.

### Limitations

We admit some limitations in our study. First, this study uses data from a single platform, YiDianLing, collected at 4 intervals throughout the year. While YiDianLing is a reliable source, relying on one website may limit the generalizability of our findings. The 4 data collection periods were chosen to cover key times of the year, providing a representative snapshot. However, future research should incorporate data from multiple platforms and extend the observation period to enhance the findings’ robustness and applicability. Second, although our study on online psychological counseling in China offers valuable insights, there is significant variability in counseling services across different countries, particularly in terms of providers and content. Future research should include data from multiple countries to capture a more comprehensive view of global trends and differences in online psychological counseling. Third, this study focuses on using signaling theory to explore clients’ decision-making processes when choosing online psychological counselors, emphasizing the behaviors prior to engaging in online counseling. However, psychological counseling is inherently a long-term and complex process. Future research should incorporate assessments of the long-term outcomes and effectiveness of counseling sessions to comprehensively evaluate the overall process of online psychological counseling. Additionally, the results and effectiveness of clients’ previous psychological counseling sessions may also influence their choices to some extent. Due to privacy protections, YiDianLing has not disclosed data regarding these past experiences. Future research should consider addressing this limitation by finding ways to incorporate such data while ensuring privacy protection. Fourth, while we considered regional diversity in our sample selection and controlled for the city tiers of psychological counselors in our data analysis, the lack of direct data on clients’ locations may impact the generalizability of our results. Under the condition of complying with relevant regulations, future research should collect and analyze data on clients’ locations to enhance the study’s comprehensiveness. Fifth, counselors’ home pages also contain some textual data that disclose clients’ experiences, but this paper only analyzed some quantitative data and future research can do deeper mining of textual data of OHCs.

### Conclusions

This study uses signaling theory to investigate client preferences for psychological counselors. We segmented the information displayed on counselors’ home pages into online and offline signals and established a theoretical model for client decision making using data derived from a Chinese online health community. The findings reveal that service price, online reputation, professional titles, and practical experience positively influence clients’ choices. Additionally, we discovered an inverted U-shaped correlation between a psychological counselor’s prosocial behavior and clients’ choices. Fundamentally, this study unravels a complex client selection process influenced by different signals, providing valuable guidance for both researchers and practitioners.

## Abbreviations

OHC: online health community
WHO: World Health Organization

## Data Availability

The datasets generated during and analyzed during this study are available from the corresponding author on reasonable request.
